# Comparison of the Clinical Use of Macintosh and Miller Laryngoscopes for Orotracheal Intubation by Second-Month Nurse Students in Anesthesiology

**DOI:** 10.1155/2010/432846

**Published:** 2010-03-22

**Authors:** Somchai Amornyotin, Ungkab Prakanrattana, Phongthara Vichitvejpaisal, Thantima Vallisut, Neunghathai Kunanont, Ladda Permpholprasert

**Affiliations:** ^1^Department of Anesthesiology, Faculty of Medicine Siriraj Hospital, Mahidol University, Bangkok 10700, Thailand; ^2^Siriraj GI Endoscopy Center, Faculty of Medicine Siriraj Hospital, Mahidol University, Bangkok 10700, Thailand

## Abstract

*Aim*. The aim of this study is to compare the clinical feasibility of Macintosh and Miller laryngoscopes for tracheal intubation in non-experienced users in anesthetized patients. *Patients and Methods*. 119 patients were randomized into the Macintosh group (59) and the Miller group (60). The primary outcome variable was successful tracheal intubation. The secondary outcome variables were number of insertion attempt, intubation time needed, total time to intubation, hemodynamic change and complications. *Results*. All patients were successfully intubated using the Macintosh, whereas 13 patients (21.6%) were failed with the Miller (*P* < .001). The Macintosh significantly reduced the mean total time to intubation (*P* < .001). There were significant differences in the mean blood pressure at 2 minutes after laryngoscope insertion, immediately, and 2 minutes after tracheal intubation and in the mean heart rate at the laryngoscope insertion, immediately, and at 2 minutes after tracheal intubation between the two groups. Overall complications in both were not significantly different. *Conclusion*. Orotracheal intubation using the Macintosh is an effective and safe technique in non-experienced hands with significantly increased success rate as well as decreased mean total time to intubation as compare to the Miller. However, these intubations only apply to selected patients deemed to have normal airways.

## 1. Introduction

Tracheal intubation is the most important and safest technique used to provide a definite airway whenever controlled ventilation is required. Most routine orotracheal intubation is performed with the help of a laryngoscope [1–3]. Either Macintosh or Miller laryngoscope is used by most anesthesia personnel for laryngoscopy and tracheal intubation. 

 Many anesthetic personnel traditionally learn how to perform laryngoscopy and intubation using the Macintosh laryngoscope. Additionally, many anesthetic personnel perform laryngoscopy with the Miller laryngoscope in the same way they would using a curved blade. Laryngoscopic competency in tracheal intubation is essential for many healthcare trainees. Failure to perform successful intubation can sometimes result in patient death. Traditional teaching on this matter has focused on certain key aspects of successful intubation, including the technical details of proper head positioning with laryngoscope blade insertion and lift, as well as a timely and atraumatic performance.

 This study was designed to compare the clinical feasibility of Macintosh and Miller laryngoscopes in the hands of inexperienced users in anesthetized patients deemed to be at low risk of difficult intubation. We hypothesized that both devices perform similarly, concerning success of an endotracheal intubation. Additionally, we studied the number of insertion attempts, time needed for insertion, hemodynamic changes, and complications. 

## 2. Materials and Methods

### 2.1. Instruction Tool

The instructional materials included a Power Point presentation and a manikin head (Laerdal Airway Management Teacher, Laerder, Denmark) on which to practice, Macintosh and Miller laryngoscopes as well as endotracheal tube (ETT, 7.5: women, 8: men, cuffed, Protex, STMS Portex Limited, UK) for the novice.

 The presentation containing instructions and illustrations regarding tracheal intubation step by step was developed by the authors skilled in the use to both laryngoscopes, with reference to the manufacturer's instructional manual and the standard text books [2, 3].

### 2.2. Patients

The study was conducted from December 2005 to March 2006 at a large tertiary care referral center, Siriraj Hospital, Bangkok, Thailand. Patients with age at least 18 years of age who scheduled for elective surgical procedures requiring tracheal intubation were eligible for the study. The exclusion criteria were risk factors for gastric aspiration and/or difficult intubation (Mallampati class III or IV; thyromental distance less than 6 cm; interincisor distance less than 4 cm) and history of relevant drug allergy. The study was approved by the Institutional Review Board of the Faculty of Medicine Siriraj Hospital. All patients provided written informed consent for the study and the procedure.

### 2.3. Study Design

The study is a clinical trial randomized control study. Patients were randomized into either Macintosh laryngoscope (MC) group or Miller laryngoscope (ML) group by using computerized generated randomization numbers placed in sealed envelopes. General anesthesia with endotracheal tube requiring muscle relaxation was performed in the operating room. Successful tracheal intubation was the primary outcome measured. Failure to success was defined as an inability to place an endotracheal tube after three attempts or a significant alteration of hemodynamic (severe hypertension and serious cardiac arrhythmia) and respiratory parameters (O_2_ saturation < 90%) during intubation or an inevitable intervention of staff anesthesiologists. Secondary outcome variables were number of insertion attempts, intubation time needed (insertion of the laryngoscope into the oropharynx to the time of its removal), total time to intubation (the sum of the durations of all (as many as three) intubation attempts), hemodynamic change, and complications.

### 2.4. Tracheal Intubation Technique

Thirty nurse students in anesthesiology without intubation experience followed the instruction on the use of Macintosh and Miller laryngoscopes. Standard monitoring, including electrocardiography, noninvasive blood pressure, oxygen saturation, and end tidal carbon dioxide were continuously performed. After preoxygenation with 100% oxygen for 3 minutes, anesthesia was induced with fentanyl 1 mcg/kg followed by propofol 2-3 mg/kg. The patients' lungs were manually ventilated with isoflurane (1.5–2.0%) in oxygen; pancuronium 0.08–0.1 mg/kg was administered. The patient's trachea was intubated at 3 minutes after the induction of anesthesia. Anesthesia was maintained with fentanyl, pancuronium, and isoflurane (0.75–1.0%) in a mixture of nitrous oxide and oxygen 2 : 1. 

### 2.5. Intubation Assessment

The success rate of tracheal intubation in both groups was evaluated. The number of insertion attempts, intubation time needed, total time to intubation, hemodynamic change, and complications was also recorded. The intubation time defined as the time needed from insertion of the laryngoscope into the oropharynx to the time of its removal. The total time to intubation was the sum of the durations of all (as many as three) intubation attempts.

### 2.6. Statistical Analysis

Results were expressed as mean±SD or percentage (%), when appropriate. Comparisons between Macintosh laryngoscope and Miller laryngoscope groups were compared by using with Chi-square tests (for categorical variables), Chi-square tests for trend (for ordinal variables), and two-sample independent *t*-test (for continuous variables). The statistical software package SPSS for Window Version 11 (SPSS Inc., Chicago, IL) was used to analyze the data. All statistical comparisons were made at the two-sided 5% level of significance.

## 3. Results

Of the total 119 patients randomized, 59 patients were randomized to group MC while 60 patients to group ML. Table 1 summarized the clinical characteristics of the two groups. The mean ages in both groups were similar: 41.8 ± 7.9 (range: 22–62) years in group MC and 42.5 ± 11.2 (range: 18–64) years in group ML (*P* = .566). There were no statistically significantly differences in gender, age, weight, height, body mass index, ASA physical status, and Mallampati score between the two groups. 

 Overall success rate, intubation attempt, intubation time, and the lowest oxygen saturation during intubation attempt are shown in Table 2. All patients in the Macintosh group were successfully intubated on the first attempt whereas 13 patients (21.6%) in the Miller group were failed (*P* < .001). All failed intubated cases were successfully intubated by using the Macintosh laryngoscope. Intubation time in group MC was significantly shorter than in group ML (*P* < .001). The lowest oxygen saturation during intubation attempt in both groups was 98% (*P* = .929).

Table 3 showed the hemodynamic parameters and oxygen saturation during and immediately after tracheal intubation. Mean systolic blood pressure throughout the study was not statistically different between the two groups except at 2 minutes after the laryngoscope insertion and at 2 minutes after the tracheal intubation. Additionally, mean diastolic blood pressure was not statistically different between the two groups except at 2 minutes after the laryngoscope insertion and at immediately after tracheal intubation. Furthermore, there were significant differences in heart rate at the laryngoscope insertion, immediately after tracheal intubation, and at 2 minutes after tracheal intubation. Mean SpO_2_ of all patients was 99% throughout the study.

 Procedure related complications during and immediately after tracheal intubation were demonstrated in Table 4. The overall complications occurred in two patients (3.4%) in group MC and eight patients (13.3%) in group ML (*P* = .435). All complications were mild degree and did not require any specific interventions including oral mucosa laceration 3.4% in group MC and 6.7% in group ML; bleeding, none in group MC and 5.0% in group ML; dental injury, none in group MC and 1.7% in group ML.

## 4. Discussion

 This prospective randomized study compared clinical performances of two laryngoscopes in the hands of inexperienced users. Our study demonstrated that the Macintosh provides superior intubating conditions in the normal airway with regards to successful intubation, as judged by achieving adequate ventilation within three insertion attempts. All tracheal intubations in the Macintosh group were successful in the first attempt; whereas 13 patients (21.6%) in the Miller group were failed. In additional, the failed intubations in the Miller group could be successfully intubated by using the Macintosh laryngoscope.

 Orotracheal intubation by using the Macintosh, the tip of this blade should be placed into the angle made by the epiglottis with the base of the tongue. Elevation of the laryngoscope pushes the base of the tongue upward; whereas the epiglottis is drawn upward, providing a clear view of the larynx. On the other hand, the Miller blade is straight; however, the tip should extend just behind (posterior to) or beneath the laryngeal surface of the epiglottis [1–4]. As with the both, subsequent forward and upward movement of the blades exposes the glottic opening. The Macintosh is also thought to be less traumatic to the teeth and to provide more room for passage of the tracheal tube through the oropharynx. However, the Miller provides a better view of the glottis in a patient with a long, floppy epiglottis, or an anterior larynx. Therefore, this laryngoscope is preferred in infants, pediatric patients, and patients with an anterior larynx [1]. Arino et al. [5] reported that a good laryngeal view with the intubating device did not equate with ease of intubation. Since the most important aspect of a laryngoscopic intubation is the correct placement of the tracheal tube, and not the visualization of the larynx.

 Many types of laryngoscopes have been used for tracheal intubation [5–9]. In developing countries like Thailand, direct laryngoscopy for tracheal intubation is usually performed with the Macintosh laryngoscope. However, direct laryngoscopy and intubation is a medical procedure that requires an experience [10]. Additionally, the novice's experience is one of the most successful intubation factors [11]. The general nurses in our hospital and in other hospitals in Thailand are not trained to perform laryngoscopy and tracheal intubation. However, the nurse students in anesthesiology are trained to perform it. The community hospitals in developing country like Thailand have none or few anesthesiologists. Laryngoscopy and tracheal intubation for general anesthesia is done by anesthetic nurses. Although our nurse students in anesthesiology felt familiar with the laryngoscopes after instruction and training on a manikin, their experiences remained limited compared to the clinicians. The limited experience may also explain the significant difference in success rate between the two groups. To exclude this in our study, we chose to compare clinical experiences of both laryngoscopes in the hands of second-month nurse students in anesthesiology. 

 The Macintosh resulted in less stimulation of systolic and diastolic blood pressure and heart rate following tracheal intubation in comparison with the Miller laryngoscope, but not significantly different. The hemodynamic findings for direct laryngoscopy in our study were similar to those described previously [12–14].

 The reported incidences of complications in both groups were very low. As might be expected in this study of patients at low risk for difficult laryngoscopy, there were no incidences of serious complications with either laryngoscope.

 Our study has some limitations in many respects. First, as data were collected unblinded, some bias is possible. Second, there is no well-defined, acceptable to total time to intubation in the literature. As a consequence, for the purpose of this study, successful tracheal intubation was defined as either an ability to place the endotracheal tube within three attempts or, there was a nonsignificant alteration of the hemodynamic and respiratory parameters. Third, the traditionally laryngoscopic tracheal intubation teaching for nonanesthesia personnel using manikin alone is a controversy [12]. Lastly, the novice users routinely intubated the anesthetized patients by using a Macintosh laryngoscope during the study period. They gained more experiences with the Macintosh than the Miller.

 This study shows that novice laryngoscope users could successfully intubate a patient's trachea after viewing a Power Point presentation and a manikin practicing. Orotracheal intubation by using the Macintosh laryngoscope is an effective and safe technique in nonexperienced hands with significantly increased success rate and decreased mean total time to intubation as compare to the Miller laryngoscope. However, the results of our study only apply to the selected patients deemed to have normal airways.

## Figures and Tables

**Table 1:** Patients' characteristics (mean, SD, and percentage).

	Macintosh	Miller	*P*-value
	(*n* = 59)	(*n* = 60)	
Age (yr) (mean, SD)	41.8 (7.9)	42.5 (11.2)	.566
Gender (%):			
Male	12 (20.3)	11 (18.3)	.782
Female	47 (79.7)	49 (81.7)	
Weight (kg) (mean, SD)	55.2 (10.3)	53.3 (7.4)	.105
Height (cm) (mean, SD)	154.3 (6.0)	155.8 (6.4)	.448
Body mass index (kg/m^2^) (mean, SD)	23.2 (4.0)	22.0 (3.1)	.443
ASA physical status (%):			
I	40 (67.8)	38 (63.3)	.608
II	19 (32.2)	22 (36.7)	
Mallampati score (%):			
1	42 (71.2)	45 (75.0)	.639
2	17 (28.8)	15 (25.0)	

**Table 2:** Overall success rate, intubation attempt, intubation time, and the lowest SpO_2_ during intubation attempt.

	Macintosh	Miller	*P*-value
	(*n* = 59)	(*n* = 60)	
Overall success rate (%)	59 (100.0)	47 (78.4)	<.001*
Intubation attempt (%)			.037*
1	59 (100.0)	42 (70.0)	
2	0	4 (6.7)	
3	0	1 (1.7)	
Intubation time (sec) (mean, SD)	25.4 (10.7)	46.9 (17.7)	<.001*
Lowest SpO_2_ during intubation attempts (mean, SD)	98.3 (0.6)	98.0 (0.6)	.929

*Considered statistically significant.

**Table 3:** Hemodynamic parameters: systolic and diastolic blood pressure (mmHg), heart rate (beat/minute), and oxygen saturation (SpO_2_, %) (mean, SD).

	Macintosh	Miller	*P*-value
	(59)	(47)	
Baseline			
SBP, DBP	121.4 (11.9), 71.2 (9.6)	122.4 (16.5), 70.5 (13.4)	.113, .082
HR, SpO_2_	81.0 (10.9), 99.8 (0.4)	76.0 (11.5), 99.7 (0.6)	.148, .129

At insertion			
SBP, DBP	131.0 (19.1), 73.2 (12.0)	123.0 (20.0), 71.2 (14.0)	.145, .104
HR, SpO_2_	80.5 (13.2), 99.8 (0.4)	78.3 (11.7), 99.9 (1.9)	.036*, .088

1 min			
SBP, DBP	129.7 (18.1), 73.7 (10.5)	122.4 (16.0), 72.1 (12.2)	.270, .166
HR, SpO_2_	80.2 (10.4), 99.8 (0.5)	79.6 (13.2), 99.6 (0.8)	.130, .361

2 min			
SBP, DBP	130.0 (16.4), 77.3 (9.4)	123.8 (17.2), 73.8 (11.1)	.033*, .032*
HR, SpO_2_	82.9 (10.0), 99.7 (0.7)	78.0 (11.6), 99.6 (0.7)	.088, .510

3 min			
SBP, DBP	121.5 (12.6), 71.2 (9.3)	121.4 (16.6), 73.2 (9.7)	.315, .086
HR, SpO_2_	80.5 (9.9), 99.7 (0.5)	77.6 (8.7), 99.7 (0.6)	.069, .610

Immediately after TT			
SBP, DBP	131.4 (15.8), 76.6 (10.2)	132.8 (14.9), 85.6 (9.0)	.709, .024*
HR, SpO_2_	85.1 (6.3), 99.6 (0.6)	93.7 (7.5), 99.6 (0.6)	.013*, .929

2 min after TT			
SBP, DBP	120.8 (14.6), 74.4 (11.2)	132.6 (17.7), 77.2 (11.2)	.019*, .244
HR, SpO_2_	84.3 (10.0), 99.6 (0.7)	82.6 (8.6), 99.8 (0.4)	.007*, .323

4 min after TT			
SBP, DBP	121.3 (17.3), 73.0 (11.0)	128.7 (17.7), 77.1 (7.6)	.144, .172
HR, SpO_2_	83.5 (10.2), 99.8 (0.4)	80.7 (7.0), 99.7 (0.4)	.142, .807

6 min after TT			
SBP, DBP	118.3 (16.4), 70.5 (11.4)	123.7 (12.9), 76.3 (8.2)	.352, .196
HR, SpO_2_	83.4 (9.5), 99.8 (0.5)	79.7 (7.4), 99.8 (0.4)	.087, .517

8 min after TT			
SBP, DBP	117.6 (14.5), 69.6 (9.7)	122.5 (10.0), 76.3 (6.5)	.432, .231
HR, SpO_2_	83.2 (8.3), 99.7 (0.7)	81.9 (6.2), 99.8 (0.4)	.162, .166

10 min after TT			
SBP, DBP	120.6 (17.1), 71.9 (12.3)	113.2 (31.4), 73.3 (9.1)	.175, .181
HR, SpO_2_	84.1 (7.5), 99.8 (0.5)	84.1 (7.5), 99.8 (0.5)	.177, .763

SBP: Systolic blood pressure; DBP: Diastolic blood pressure; HR: Heart rate; SpO_2_: Oxygen.

Saturation; TT: Tracheal intubation.

*Considered to be of statistical significance.

**Table 4:** Procedure related complications during and immediately after tracheal intubation (*n*, %).

	Macintosh	Miller	*P*-value
	(*n* = 59)	(*n* = 60)	
Overall	2 (3.4)	8 (13.3)	.435
Oral mucosa laceration	2 (3.4)	4 (6.7)	.414
Bleeding	0	3 (5.0)	.082
Dental injury	0	1 (1.7)	.319

*Considered to be of statistical significance.
